# Influence of autolyzed whole yeast and yeast components on broiler chickens challenged with salmonella lipopolysaccharide

**DOI:** 10.3382/ps/pez452

**Published:** 2019-08-08

**Authors:** E U Ahiwe, M E Abdallh, E P Chang'a, M Al-Qahtani, A A Omede, H Graham, P A Iji

**Affiliations:** 1 School of Environmental and Rural Sciences, University of New England, Armidale NSW 2351 Australia; 2 Department of Animal Science and Technology, Federal University of Technology, Owerri PMB 1526, Imo State, Nigeria; 3 Department of Poultry Production, University of Khartoum, Khartoum 13314, Sudan; 4 Tanzania Livestock Research Institute (TALIRI), P. O. Box 352, Mwanza, Tanzania; 5 Department of Animal Production, Kogi State University, Anyigba PMB 1008, Kogi State, Nigeria; 6 AB Vista UK, Marlborough, Wiltshire SN8 4AN, UK; 7 College of Agriculture, Fisheries and Forestry, Fiji National University, Koronivia 1544, Fiji

**Keywords:** broiler chickens, yeasts, antibiotics, mild stress, gross response

## Abstract

The objective of this study was to assess the effect of dietary yeast products on broiler chickens challenged with salmonella lipopolysaccharide (**LPS**). The chicks were divided into 8 treatments with 6 replicates and 9 birds per replicate. The treatments consisted of a positive control (**PC**) [without supplementation and not challenged]; negative control (**NC**) [without supplementation but challenged]; whole yeast and challenged; yeast cell wall and challenged; yeast glucan and challenged; yeast mannan and challenged; zinc bacitracin and challenged; and Salinomycin and challenged. Whole yeast or Yeast cell wall was included at 2.0 g/kg diet. Yeast glucan or mannan was added at 0.20 g/kg diet. Zinc bacitracin (**ZNB**) and Salinomycin (**SAL**) was included at 50 and 60 ppm, respectively. Dietary treatments had no effect (*P* > 0.05) on feed intake (**FI**) at day 10. Supplementation with yeast and its derivatives improved (*P* < 0.05) body weight gain (**BWG**) and feed conversion ratio (**FCR**) on day 10. On days 24 and 35, LPS challenge declined FI, BWG, FCR, and flock uniformity (day 28) in the NC group compared to the PC group. Yeast products and antibiotics improved (*P* < 0.05) FI, BWG, FCR, and flock uniformity in LPS-challenged birds. On day 24, spleen weight increased while bursa weight decreased in the NC group relative to the PC group; this effect was reversed (*P* < 0.05) by feeding all yeasts and antibiotics. On day 24, application of all the dietary treatments ameliorated the changes observed in white blood cell, lymphocyte and monocyte counts as well as albumin and immunoglobulin G of NC birds. On day 35, all yeasts additives, ZNB and SAL improved (*P* < 0.05) the meat yield of broilers challenged with LPS. In conclusion, supplementation of diets with yeast and its derivatives can ameliorate the negative effects of salmonella LPS challenge on broiler chicks, thus improving the performance, flock uniformity, and meat yield.

## INTRODUCTION

Antibiotics have been routinely supplemented in diets of intensively reared broiler chickens to maintain their health, reduce stress, and enhance productivity (Engberg et al., 2000; Paradis et al., 2016). However, due to the development of resistance of bacteria to antibiotics and the possibility of these pathogens to be zoonotic, the use of various antibiotic products in livestock and poultry production is gradually being banned around the world (Chang et al., 2014). With the possibility of further ban in more regions of the world, research interest into alternatives to in-feed antibiotics has increased (Zhang and Kim, 2014).

One alternative to in-feed antibiotics that has gained research interest for use in broiler diets is yeast (*Saccharomyces cerevisiae*) and its components: yeast cell wall (**YCW**), yeast mannan (**YM**), or yeast glucan (**YG**). Yeast in both probiotic (live) and prebiotic (dead) forms has been reported to provide several benefits to both healthy and disease-challenged animals, including poultry (Onwurah et al., 2014; Hana et al., 2015). The ability of yeast and its components to act as growth promoter, as well as disease and stress ameliorators could be associated with different mechanisms that it exhibits individually or synergistically. For instance, yeasts have been reported to favor the proliferation of beneficial microbes by serving as substrates for these microbes in the gut (Pan and Yu, 2013). These beneficial microbes, such as *Lactobacillus*, have been reported to improve gut health as well as exhibit growth-promoting effects in broiler chickens. Specifically, yeast cell wall that is extracted from whole yeast consists mainly of α-mannans and β-1–3-glucans, which are reported to prevent or eliminate bacterial infections (Baurhoo et al., 2012).

To the best of our knowledge, little research has been conducted to assess the role of autolyzed (prebiotic) yeast and its enzymatically hydrolyzed components in relation to antibiotics or coccidiostat in reducing the impact of mild disease or stress challenge in broiler chickens reared in a controlled environment. Furthermore, this study provided an opportunity to test 4 novel products of yeast origin along with 2 antibiotic products, zinc bacitracin and Salinomycin supplemented in diets on the gross and mechanistic responses of broiler chickens under salmonella lipopolysaccharide (**LPS**) challenge.

## MATERIALS AND METHODS

This experiment was approved by the Animal Ethics Committee of the University of New England (Approval number: AEC17–115). Health and animal husbandry practices complied with the code of practice for the use of Animals for Scientific purpose issued by the Australian Bureau of Animal Health.

### Diets, Experimental Design, and Feeding

Eight basal diets, consisting mainly of corn and soybean, were used in a 3-phase feeding programme, with the starter feed (crumbles) provided from days 0 to 10, grower feed (pelleted) from days 11 to 24 and finisher feed (pelleted) from days 25 to 35 of age (Table 1). Most of the ingredients were purchased from a local supplier in northern New South Wales, Australia. Experimental diets, were formulated to Aviagen standards for Ross 308 (Aviagen, 2014). The experiment had 8 treatments, each treatment having 6 replicates of 9 birds each. The eight treatments consisted of a positive control (**PC**) [without supplementation and not challenged]; negative control (**NC**) [without supplementation but challenged with salmonella LPS] at days 13, 15, and 17; whole yeast (added at 2.0 g/kg diet) and also challenged with LPS (**WYC**); yeast cell wall (added at 2.0 g/kg diet) and challenged (**YCWC**); yeast glucan (added at 0.20 g/kg diet) and also challenged with LPS (**YGC**); yeast mannan (added at 0.20 g/kg diet) and also challenged with LPS (**YMC**); zinc bacitracin (added at 50 ppm) and also challenged with LPS (**ZNBC**); and Salinomycin (added at 60 ppm) and also challenged with LPS (**SALC**). The additives were included according to manufacturer's recommendation, and have been previously used at the same levels for healthy chickens with excellent results (Ahiwe et al., 2018; Ahiwe et al., 2019). Feed and water were offered *ad libitum*.

**Table 1.** Ingredient and nutrient composition of basal diet.

	Feeding period (days)		
	Starter diet	Grower diet	Finisher diet
Ingredient (g/kg)	(0 to 10)	(11 to 24)	(25 to 35)
Corn	613.0	645.0	696.5
Soybean meal	315.0	273.5	231.3
Meat and bone meal	45.0	41.0	31.4
Canola oil	44.0	17.0	23.0
Limestone	7.7	7.1	7.1
Dicalcium phosphate	1.3	0.1	0.1
Quantum Blue 5 G (100 g)	0.1	0.1	0.1
Sodium chloride	1.0	1.1	1.4
Sodium bicarbonate	1.3	1.3	1.3
Choline Cl 70%	1.1	1.3	1.1
L-lysine HCl	3.0	2.7	2.5
DL-methionine	3.7	2.0	1.8
L-threonine	1.8	1.4	1.1
Econase GT	0.1	0.1	0.1
^1^UNE Vitamins	0.6	0.6	0.6
^2^UNE minerals	0.8	0.8	0.8
*Nutrient composition* (g/kg)			
G.E (MJ/kg)	12.6	13.0	13.4
Crude protein	230	212	192
Crude fat	28.0	39.8	45.4
Crude fibre	20.9	24.0	23.1
Lysine	12.8	11.5	10.2
Methionine	6.60	4.70	4.30
Met + Cys	9.50	7.43	6.86
Calcium	9.60	8.70	7.80
Phosphorus available	4.80	4.40	3.90
Sodium	1.60	1.60	1.60
Potassium	9.30	8.50	7.60
Chloride	2.30	2.30	2.30
Choline	1.30	1.30	1.30

^1^Vitamin premix supplied per 0.6 kg/tonne of diet: retinol, 12,000 IU; cholecalciferol, 5,000 IU; tocopheryl acetate, 75 mg; menadione, 3 mg; thiamine, 3 mg; riboflavin, 8 mg; niacin, 55 mg; pantothenate, 13 mg; pyridoxine, 5 mg; folate, 2 mg; cyanocobalamin, 16 μg; biotin, 200 μg; cereal-based carrier, 149 mg.

^2^Minerals premix supplied per 0.8 kg/tonne of diet: mineral oil, 2.5 mg; Cu (sulphate), 16 mg; Fe (sulphate), 40 mg; I (iodide), 1.25 mg; Se (selenate), 0.3 mg; Mn (sulphate and oxide), 120 mg; Zn (sulphate and oxide), 100 mg; cereal-based carrier, 128 mg; mineral oil, 3.75 mg.

### Birds, Housing, and Management

A total of 432-day-old Ross 308 male broiler chicks (initial weight, 40.4 ± 1.02 g) obtained from a local hatchery (Baiada Poultry PTY. Ltd, Tamworth, NSW, Australia) were randomly assigned to 48 pens bedded with fresh wood shavings in an environmentally controlled room. Each pen was equipped with a feeder and nipple drinkers. Average BW and uniformity were considered when weighing and allocating the nine chicks into each pen (80 × 47 × 45 cm) containing the dietary treatments. Initial brooding temperature was 33°C, but this was gradually reduced to 24 ± 1°C by 19 d of age. The lighting programme was 18 h light and 6 h darkness throughout the trial.

### Productive Performance Measurement, Administration of LPS, and Temperature Check

**Figure 1.**
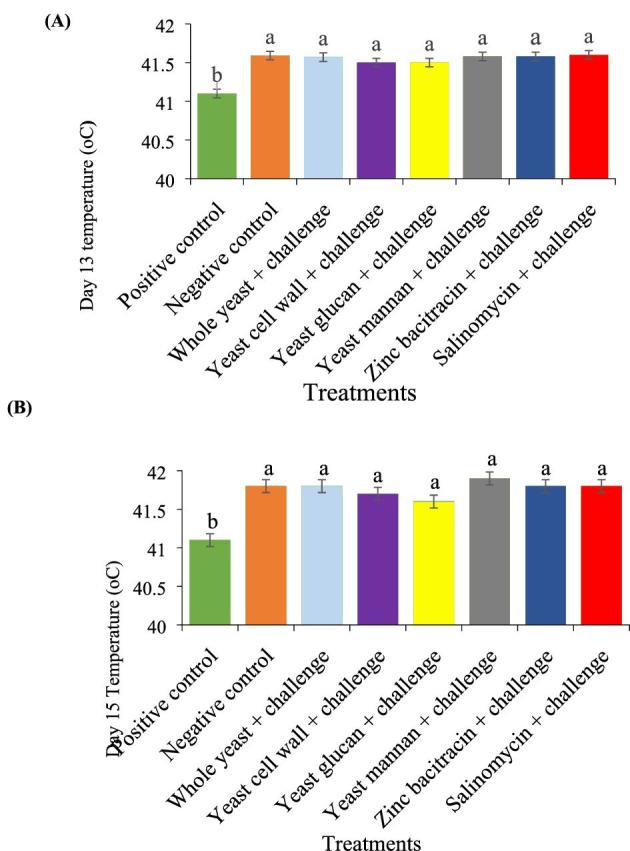
Three hours post-administration of *Salmonella* lipopolysaccharide temperature changes [(A) day 13 and (B) day 15] of broiler chickens fed diets with or without the dietary treatments. Data were presented with mean ± SE (n = 6 for each mean). Different letters indicates significant differences (*P* < 0.05).

Body weight (**BW**) and feed intake (**FI**) were recorded on days 10, 24, and 35. Mortality was recorded daily and feed per gain values were corrected for mortality. On days 13, 15, and 17, the birds on the challenged treatments were injected intraperitoneally with 3 ml of a solution of LPS (from *Salmonella enterica* serotype Typhimurium, Sigma Chemical Co., St. Louis, Mo 63,178–9916) made up as 100 *µ*g/ml in 0.9% saline. The cloacal temperature of the bird was measured by inserting a thermocouple probe (Physitemp Instruments Inc., Clifton, New Jersey, USA) 5 cm into the cloaca of one bird per replicate 4 h after injections on days 13 and 15, to confirm reaction to the treatment. The administration of LPS led to an increase in temperature in all treatments (Figure 1).

### Sampling Procedures and Measurements

#### Visceral Organ, Blood Collection, and Meat Yield Sampling

At days 10 and 24, one bird per cage (6 birds per treatment) was electrically stunned, killed by cervical dislocation, and key visceral organs (gizzard together with the proventriculus, heart, small intestine, pancreas, liver, spleen, and bursa) excised and weighed. The relative weight of these organs was estimated as mass per unit of live BW (g/kg of live BW). At the end of the experimental period (day 35), 2 birds per replicate were randomly selected from each pen, weighed and euthanized by electrically stunning them, followed by cervical dislocation. After de-feathering, the breast (pectoralis minor and pectoralis major), thighs and drumsticks (bones included) were separated from the carcass and weighed. The relative parts weight was calculated as mass per unit live BW (g/kg of live BW). On day 24, blood samples were collected from two birds per pen. The birds were electrically stunned and their jugular vein cut to allow blood flow. The blood samples were allowed to flow into tubes coated with EDTA, and used to determine haematological parameters in whole blood and plasma.

### Flock Uniformity Calculation; Haematological Indices and Plasma Metabolites Determination

The birds (5 remaining birds) were individually weighed at day 28 of age. Bird uniformity was calculated from the coefficient of variation of their individual body weight according to the following equation as described by Jackson et al. (2004).

$$
\begin{eqnarray*}
&&{\rm{Flock}}\,{\rm{uniformity}}\nonumber\\
&&\quad = {\rm{100}}-\left( {\left( {\frac{{ABW}}{{STDEV}}} \right) \times {\rm{100}}} \right)
\end{eqnarray*}$$

where ABW = Average live body weight and STDEV = standard deviation of a single replicate.

Cell counts (white blood cells, red blood cells, heterophils, lymphocytes (**LYMs**), monocytes (**MONOs**), eosinophils, and basophils), haemoglobin content, mean corpuscular volume and haematocrit of blood samples were determined using an automated haematological analyzer (Cell-Dyn 3700, Abbott Laboratories, Abbott Park, IL, USA). A sandwich ELISA protocol according to the method of Schuijffel et al. (2005), was used to determine the concentration of chicken plasma total protein, albumin, globulin, cholesterol, triglyceride, high-density lipoprotein cholesterol, and low-density lipoprotein cholesterol contents were measured using a clinical analyzer (Siemens Dimension Xpand, Australia).

### Statistical Analysis

All data were subjected to analysis of variance (ANOVA) in the general linear model of Minitab 17 statistical package (Minitab, 2013). Separations of means within a significant effect were carried out using the Tukey Range Test, while differences between treatments were compared using planned orthogonal probability contrast. Significance levels were set at *P* ≤ 0.05.

## RESULTS

### Growth Performance

The effect of experimental treatments on performance of broilers at different ages is summarized in Table 2. On day 10 (pre LPS administration), none of the dietary treatments had a significant effect (*P* > 0.05) on FI. However, broilers in the WYC, YCWC, YMC, ZNBC, and SALC groups had better (*P* < 0.05) body weight gain (**BWG**) and feed conversion ratio (**FCR**) compared to broilers in the PC and NC groups, while broilers in the YGC group were intermediate.

**Table 2.** Effects of dietary treatments on growth performance of broiler chickens at different ages.^1^

	Day 10			Day 24			Day 35		
	FI	BWG	FCR	FI	BWG	FCR	F1	BWG	FCR
	(g/bird)	(g/bird)	(g/g)	(g/bird)	(g/bird)	(g/g)	(g/bird)	(g/bird)	(g/g)
PC	302.6	267.9^c^	1.13^a^	1683.0^a^	1373.7^a^	1.23^b^	3416.3^a^	2354.9^a,b^	1.45^b^
NC	304.3	269.9^c^	1.12^a^	1618.7^b^	1226.4^c^	1.33^a^	3303.2^b^	2191.0^c^	1.51^a^
WYC	293.5	292.9^a,b^	0.99^b^	1654.2^a,b^	1344.2^a,b^	1.23^b^	3391.2^a,b^	2342.0^a,b^	1.45^b^
YCWC	293.7	291.1^a,b^	0.99^b^	1669.8^a,b^	1351.2^b^	1.24^b^	3418.1^a^	2360.7^a,b^	1.45^b^
YGC	288.7	277.7^b,c^	1.03^a,b^	1648.1^a,b^	1343.3^a,b^	1.24^b^	3355.9^a,b^	2298.7^b^	1.46^b^
YMC	291.7	287.4^a,b^	0.99^b^	1641.2^a,b^	1338.3^a,b^	1.24^b^	3383.1^a,b^	2329.6^a,b^	1.45^b^
ZNBC	298.3	299.2^a^	0.99^b^	1673.1^a^	1374.9^a^	1.22^b^	3382.7^a,b^	2399.1^a^	1.41^b^
SALC	294.2	293.9^a,b^	1.00^b^	1646.4^a,b^	1341.8^a,b^	1.22^b^	3363.0^a,b^	2351.4^a,b^	1.43^b^
SEM	2.16	2.20	0.01	5.53	9.15	0.01	8.79	11.1	0.01
*P*-value	0.63	0.001	0.01	0.02	0.001	0.02	0.02	0.001	0.001
	Probability level of orthogonal contrasts								
NC vs PC	0.90	0.78	0.93	0.01	0.01	0.01	0.001	0.03	0.006
NC vs WYC	0.31	0.003	0.04	0.07	0.03	0.01	0.04	0.02	0.001
NC vs YCWC	0.38	0.003	0.04	0.08	0.02	0.01	0.001	0.01	0.001
NC vs YGC	0.23	0.23	0.13	0.44	0.04	0.02	0.06	0.02	0.003
NC vs YMC	0.35	0.02	0.04	0.14	0.04	0.01	0.01	0.03	0.001
NC vs ZNBC	0.13	0.001	0.006	0.02	0.03	0.01	0.03	0.008	0.001
NC vs SALC	0.42	0.005	0.04	0.19	0.03	0.01	0.04	0.01	0.001

a–c Means in a column not sharing a common superscript differ (*P* < 0.05).

1 Values are means of 6 replicates; NC = Negative control; PC = Positive control; WYC = Whole Yeast + LPS challenge; YCWC = Yeast cell wall extract + LPS challenge; YGC = Yeast glucan extract + LPS challenge; YMC = Yeast mannan extract + LPS challenge; ZNBC = Zinc bacitracin + LPS challenged; SALC = Salinomycin + LPS challenged. FI = Feed intake; BWG = Body weight gain; FCR = Feed conversion ratio.

On day 24 (post LPS administration), there was a decrease (*P* < 0.05) in FI in the NC group compared to the PC and ZNBC groups, with other groups being intermediate. LPS challenge resulted in a decrease (*P* < 0.05) in BW of broilers in the NC group relative to other groups. It was also noted that broilers in the NC group had the poorest (*P* < 0.05) FCR compared to other experimental groups.

At the end of the experiment (day 35), broilers in the NC group had lower (*P* < 0.05) FI compared to broilers in the PC and YCWC groups, while broilers in the other groups were intermediate. Birds in the NC group had poorer (*P* < 0.05) BWG and FCR compared to broilers in the PC group. However, the addition of WY, YCW, YG, YM, ZNB, and SAL resulted in an amelioration (*P* < 0.05) of the negative effect of LPS on BWG and FCR noticed in the NC group.

All through the experiment, there was no significant (*P* > 0.05) effect of any of the test additives and LPS challenge on percentage livability of the birds (Data not shown).

### Visceral Organ Weight

Overall, the relative weight of all the visceral organs (Proventriculus + Gizzard (PGizzard), pancreas, small intestine, heart, spleen, bursa, and liver) considered on day 10 of the present study (pre-LPS administration) was not affected (*P* > 0.05) by dietary treatments (Data not shown). As shown in Table 3, on day 24 there was no significant effect (*P* > 0.05) of dietary treatment or LPS challenge on the relative PGizzard, pancreas, small intestine, heart, and liver weights. However, spleen weight increased while the bursa weight decreased in the NC group (broiler chickens challenged with LPS and un-supplemented) relative to birds in the PC group; this effect was alleviated (*P* < 0.05) by feeding all test products. Broilers in the WYC, YCWC, YGC, YMC, ZNBC, and SALC groups had comparable relative weight of spleen and bursa to broilers in the PC group.

**Table 3.** Effects of dietary treatments on visceral organ weight (g/100 g) of chicks on 24 d of age.

Item	PGizzard	Pancreas	S.1	Heart	Liver	Spleen	Bursa
PC	3.35	0.30	4.90	0.67	2.62	0.09^b^	0.22^a^
NC	3.43	0.33	4.90	0.79	3.11	0.12^a^	0.11^b^
WYC	2.25	0.31	4.84	0.74	2.57	0.09^b^	0.24^a^
YCWC	3.20	0.31	4.83	0.73	2.65	0.09^b^	0.24^a^
YGC	3.34	0.33	4.89	0.79	2.85	0.10^b^	0.21^a^
YMC	3.27	0.32	4.91	0.79	2.91	0.10^b^	0.20^a^
ZNBC	3.28	0.30	4.78	0.69	2.67	0.09^b^	0.24^a^
SALC	3.29	0.31	4.87	0.77	2.75	0.09^b^	0.21^a^
SEM	0.03	0.003	0.03	0.02	0.06	0.003	0.01
*P*-value	0.41	0.07	0.98	0.63	0.35	0.006	0.005
	Probability level of orthogonal contrasts						
NC vs PC	0.44	0.02	0.74	0.12	0.05	0.001	0.001
NC vs WYC	0.08	0.08	0.69	0.47	0.02	0.001	0.001
NC vs YCWC	0.02	0.03	0.57	0.37	0.06	0.002	0.001
NC vs YGC	0.38	0.95	0.94	0.10	0.28	0.006	0.01
NC vs YMC	0.21	0.48	0.94	0.90	0.40	0.04	0.02
NC vs ZNBC	0.15	0.02	0.38	0.18	0.07	0.002	0.001
NC vs SALC	0.18	0.05	0.83	0.68	0.13	0.001	0.01

a,b Means in a column not sharing a common superscript differ (*P* < 0.05). Values are means of 6 replicates; SEM = Standard error of mean; NC = Negative control; PC = Positive control; WYC = Whole Yeast + LPS challenge; YCWC = Yeast cell wall extract + LPS challenge; YGC = Yeast glucan extract + LPS challenge; YMPC = Yeast mannan extract + LPS challenge; ZNBC = Zinc bacitracin + LPS challenged; SALC = Salinomycin + LPS challenged. SI = Small intestine.

**Figure 2.**
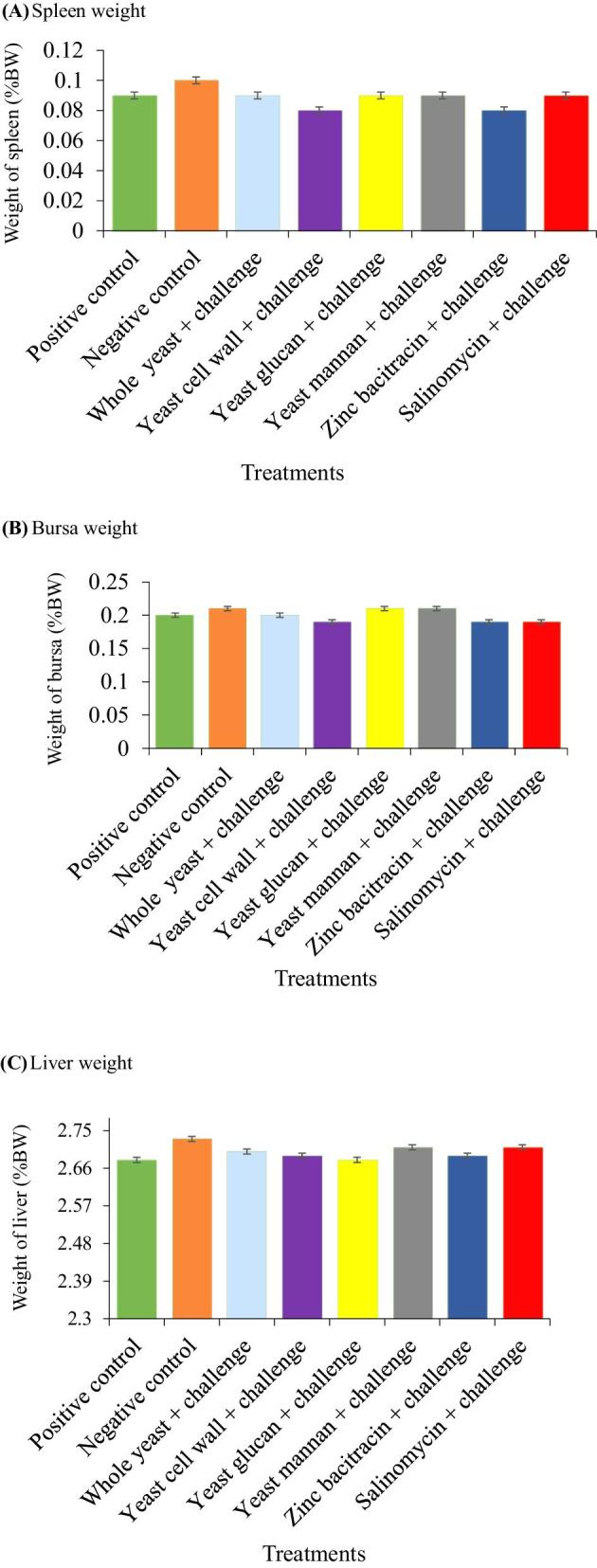
Effect of dietary treatments and LPS challenge on relative spleen, bursa and liver weight (% BW) at 35 d of age. Data were presented with mean ± SE (n = 6 for each mean).

The effect of the test additives and LPS challenge on relative weight of spleen, bursa and liver on day 35 is shown in Figure 2. None of the additives nor LPS challenge had any effect (*P* > 0.05) on the weight of the spleen, bursa or liver.

### Flock Uniformity

**Figure 3.**
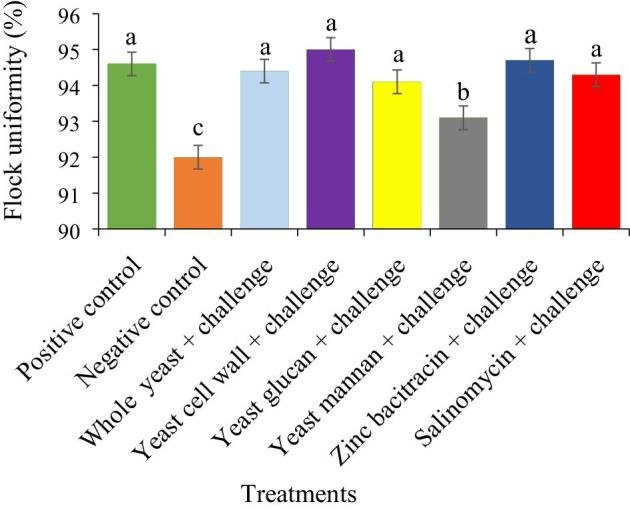
Day 28 flock uniformity (%) of broiler chickens on different dietary experimental treatments and challenged with or without LPS. Data were presented with mean ± SE (n = 6 for each mean). Different letters indicates significant differences (*P* < 0.05).

The flock uniformity of broiler chickens fed the experimental diets on day 28 is presented in Figure 3. Birds in the NC group had lower (*P* < 0.05) flock uniformity than birds in all other groups.

### Haematological Indices and Plasma Metabolites

Table 4 shows the effect of test yeast products and antibiotics on the haematological indices of broiler chickens challenged with LPS (day 24 of age). The WBC, LYM, and MONO counts were higher (*P* < 0.05) in the NC group than in the PC group. However, supplementation with whole yeast, yeast cell wall, yeast glucan, yeast mannan, and zinc bacitracin led to a modulation or decrease (*P* < 0.05) in WBC, lymphocyte and monocyte counts relative to birds in the NC group. Birds in the SALC and NC groups had similar (*P* > 0.05) WBC, lymphocyte and monocyte counts. The total protein, globulin, immunoglobulin A (**IgA**), cholesterol, triglyceride, high-density lipoprotein, and low density lipoprotein cholesterol of blood plasma at 28 d was not affected (*P* > 0.05) by dietary treatments or LPS challenge (Table 5). The albumin and immunoglobulin G (**IgG**) contents of the blood plasma in the NC group were higher than those of the PC group. However, the values of these indices for the WY, YCW, YG, YM, and ZNB groups were similar to that of the PC group while values of the SALC group were intermediate. The immunoglobulin M (**IgM**) concentration was lowest in the NC group compared to all other groups.

**Table 4.** Effects of yeast, its derivatives and antibiotics on the haematological indices of broiler chickens challenged with LPS (24 d of age).

Item	WBC	RBC	HGB	HCT	MCV	MCH	MCHC	NEU	LYM	MONO	EOS	BASO	PLT
PC	47.7^c^	2.5	12.0	27.8	114.4	53.2	44.4	12.7	61.4^b^	3.4^c^	0.04	0.78	5.8
NC	63.2^a^	2.6	11.3	27.7	114.6	52.7	46.2	13.3	76.8^a^	4.6^a^	0.04	1.01	5.9
WYC	52.1^b,c^	2.6	12.5	28.4	114.0	52.8	45.9	13.4	67.8^b^	4.0^b,c^	0.05	0.95	6.1
YCWC	52.6^b^	2.6	12.3	28.8	112.5	52.0	46.0	13.2	67.1^b^	4.0^b,c^	0.04	1.48	6.2
YGC	51.7^b,c^	2.8	12.3	28.7	112.3	51.0	45.6	13.0	66.8^b^	3.9^b,c^	0.04	1.58	6.2
YMPC	52.4^b^	2.7	12.1	28.9	113.4	51.9	45.2	13.3	66.9^b^	3.8^b,c^	0.05	1.09	6.1
ZNBC	50.1^b,c^	2.6	12.7	28.9	112.3	50.7	46.4	13.8	67.2^b^	4.0^b,c^	0.04	1.55	5.9
SALC	58.7^a,b^	2.7	12.3	28.2	114.2	52.1	45.8	13.0	76.4^a^	4.3^a,b^	0.04	0.90	6.2
SEM	0.75	0.03	0.12	0.20	0.30	0.34	0.30	0.13	0.91	0.06	0.001	0.11	0.16
*P*-value	0.001	0.78	0.57	0.40	0.25	0.64	0.77	0.51	0.001	0.001	0.88	0.32	0.80
	Probability level of orthogonal contrasts												
NC vs PC	0.001	0.40	0.64	0.93	0.86	0.71	0.13	0.25	0.001	0.001	0.98	0.58	0.91
NC vs WYC	0.001	0.69	0.14	0.40	0.59	0.95	0.78	0.89	0.003	0.002	0.89	0.88	0.79
NC vs YCWC	0.003	0.97	0.14	0.13	0.08	0.64	0.86	0.76	0.002	0.001	0.92	0.27	0.62
NC vs YGC	0.001	0.34	0.28	0.17	0.06	0.26	0.62	0.41	0.008	0.001	0.98	0.20	0.51
NC vs YMPC	0.002	0.89	0.57	0.11	0.31	0.58	0.39	0.93	0.001	0.001	0.85	0.85	0.80
NC vs ZNBC	0.001	0.85	0.06	0.10	0.06	0.16	0.90	0.34	0.09	0.001	0.97	0.17	0.92
NC vs SALC	0.09	0.72	0.29	0.61	0.75	0.69	0.72	0.45	0.82	0.13	0.98	0.79	0.64

a–c Means in a column not sharing a common superscript differ (*P* < 0.05). Values are means of 6 replicates; SEM = Standard error of mean. NC = Negative control; PC = Positive control; WYC = Whole Yeast + LPS challenge; YCWC = Yeast cell wall extract + LPS challenge; YGC = Yeast glucan extract + LPS challenge; YMPC = Yeast mannoprotein extract + LPS challenge; ZNBC = Zinc bacitracin + LPS challenged; Salinomycin + LPS challenged. WBC, White blood cells (10^6^/ml); RBC, Red blood cells (10^6^/ml); HGB, Haemoglobin (g/dl); HCT, Haematocrit (%); MCV, Mean corpuscular volume (fl); MCH, Mean corpuscular hemoglobin (pg); MCHC, Mean corpuscular hemoglobin(g/dl) NEU, Heterophils (10^6^/ml); LYM, Lymphocytes (10^6^/ml); MONO, Monocytes (10^6^/ml); EOS, Eosinophils (10^6^/ml); BASO, Basophils (10^6^/ml), PLT, Platelet (10^6^/ml).

**Table 5.** Effects of yeasts, its derivatives and antibiotics on the plasma metabolites of broiler chickens challenged with LPS (24 d of age).

				Immunoglobulin			Lipids		Lipoprotein	
	Total protein	Albumin	Globulin	(mg/ml)			(mmol/l)		(mmol/l)	
Item	(g/l)	(g/l)	(g/l)	IgA	IgG	IgM	CHOL	TG	HDL	LDL
PC	33.0	5.5^d^	26.9	0.40	8.8^c^	1.92^a^	3.4	1.4	2.5	0.61
NC	33.9	6.3^a^	27.6	0.44	9.9^a^	1.33^c^	3.5	1.4	2.7	0.62
WYC	33.7	5.9^b,c^	27.8	0.47	9.2^b,c^	1.77^a,b^	3.4	1.4	2.6	0.60
YCWC	33.5	5.7^b–d^	27.8	0.48	9.2^b,c^	1.76^a,b^	3.5	1.4	2.5	0.61
YGC	33.3	5.6^c,d^	27.7	0.47	9.1^b,c^	1.75^a,b^	3.5	1.5	2.6	0.61
YMC	33.0	5.8^b,c^	27.1	0.45	9.3^b^	1.75^a,b^	3.5	1.4	2.6	0.60
ZNBC	33.4	5.9^b,c^	27.5	0.47	9.3^b^	1.75^a,b^	3.5	1.4	2.5	0.61
SALC	33.5	6.1^a,b^	27.4	0.45	9.5^a,b^	1.60^b^	3.5	1.4	2.5	0.61
SEM	0.13	0.03	0.13	0.01	0.05	0.03	0.01	0.01	0.02	0.002
*P*-value	0.72	0.001	0.56	0.46	0.001	0.001	0.80	0.54	0.42	0.79
	Probability level of orthogonal contrasts									
NC vs PC	0.12	0.001	0.15	0.33	0.001	0.001	0.16	0.12	0.10	0.38
NC vs WYC	0.69	0.002	0.75	0.32	0.001	0.001	0.16	0.34	0.15	0.09
NC vs YCWC	0.54	0.001	0.75	0.30	0.001	0.001	0.47	0.35	0.10	0.30
NC vs YGC	0.32	0.001	0.81	0.35	0.001	0.001	0.72	0.75	0.33	0.49
NC vs YMC	0.11	0.001	0.36	0.79	0.001	0.001	0.29	0.53	0.23	0.17
NC vs ZNBC	0.37	0.001	0.84	0.36	0.003	0.001	0.28	0.21	0.34	0.22
NC vs SALC	0.47	0.06	0.71	0.75	0.09	0.001	0.28	0.22	0.34	0.38

a–d Means in a column not sharing a common superscript differ (*P* < 0.05). Values are means of 6 replicates; SEM = standard error of mean. NC = Negative control; PC = positive control; WYC = Whole yeast + LPS challenge; YCWC = Yeast cell wall extract + LPS challenge; YGC = Yeast glucan extract + LPS challenge; YMC = Yease mannan extract + LPS challenge; ZNBC = zinc bacitracin + LPS challenge; Salinomycin + LPS challenge. IG, immunoglobin, CHOL, Cholesterol; TG, Triglyceride, HDL, High-density lipoprotein cholesterol; LDL, low-density lipoprotein cholesterol.

### Carcass Yield and Meat Cut Parts

The effect of different dietary treatments on carcass dressing percentage and relative meat cut parts of broiler chickens at day 35 is presented in Table 6. The lowest (*P* < 0.05) dressing percent was recorded on the NC group. Weight of breast, thighs, and drumsticks at day 35 were depressed (*P* < 0.05) by LPS inoculation by approximately 21, 14, and 3.5%, respectively, in the NC group compared to the PC group. However, supplementation with WY, YCW, YG, YM, ZNB, and SAL improved (*P* < 0.05) the weights of the breast, thighs and drumsticks relative to the NC group.

**Table 6.** Effects of dietary treatments on dressing % and relative meat yield (g/kg carcass weight) of the chickens at 35 d of age.

Item	Dressing %	Breast	Thighs	Drumsticks
PC	76.5^a,b^	219.9^a,b^	105.5^a^	89.1^b^
NC	65.6^c^	173.0^c^	90.4^b^	85.9^c^
WYC	74.5^b^	215.2^a,b^	104.6^a^	94.5^a,b^
YCWC	77.4^a,b^	211.8^a,b^	104.6^a^	95.8^a,b^
YGC	73.8^b^	205.3^b^	101.9^a,b^	94.7^a,b^
YMPC	74.0^b^	205.9^b^	102.1^a,b^	93.6^a,b^
ZNBC	79.6^a^	232.2^a^	106.0^a^	99.8^a^
SALC	77.8^a,b^	231.2^a^	104.8^a^	98.2^a^
SEM	0.75	3.52	1.21	1.13
*P*-value	0.01	0.03	0.03	0.03
	Probability level of orthogonal contrasts			
NC vs PC	0.003	0.001	0.01	0.01
NC vs WYC	0.009	0.001	0.002	0.002
NC vs YCWC	0.003	0.001	0.002	0.001
NC vs YGC	0.02	0.001	0.01	0.006
NC vs YMPC	0.02	0.004	0.001	0.002
NC vs ZNBC	0.02	0.003	0.001	0.002
NC vs SALC	0.002	0.001	0.002	0.001

a–c Mean in a column not sharing a common superscript differ (*P* < 0.05). Values are means of 6 replicates; NC = Negative control; PC = Positive control; WYC = Whole Yeast + LPS challenge; YCWC = Yeast cell wall extract + LPS challenge; YGC = Yeast glucan extract + LPS challenge; YMPC = Yeast mannoprotein extract + LPS challenge; ZNBC = Zinc bacitracin + LPS challenged; SALC = Salinomycin + LPS challenged.

## DISCUSSION

The observed increase in cloacal temperature indicates a successful LPS challenge, and is consistent with the findings of De Boover et al. (2009), who observed a similar increase in temperature 3 h after LPS (extracted from the cell wall of *E. coli*) was intravenously administered to broilers on days 13 and 15 of age. The increase in temperature in broilers challenged with LPS relative to the PC group could be associated with the production of cytokines that lead to hypothermic and pyrogenic mechanisms that are independently activated a few hours after LPS challenge (Romanovsky et al., 1996). It was observed that none of the additives had any effect in controlling the increased temperature response 4 h after LPS administration on both days 13 and 15 of the experiment.

On day 10, birds in the WY, YCW, YM, ZNB, and SAL groups had better BWG and FCR than those in the NC and PC. The response could be linked to the supplementation of these test yeast products, which may improve gut health (through serving as substrates for the proliferation of gut health beneficial bacteria such as lactobacillus and bifidobacteria). Other possible reasons for the observed response could be the ability of the yeast products to assist in early establishment and maintenance of normal gut microflora as well as preventing the proliferation of pathogenic microbes. This action may partly contribute to increased nutrient digestibility, reduced competition for nutrients, increased nutrient utilization as well as absorption, which are associated with an improvement in BWG and FCR in broilers (Lutful Kabir, 2009). Antibiotics may improve growth of birds at an early phase through their ability to prevent the proliferation of pathogenic bacteria and maintaining a heathier poultry gut (Engberg et al., 2000).

On days 24 and 35, the poor BWG and FCR observed in broilers challenged with LPS in the NC group compared to the PC group could be associated with the decreased FI as a result of the stress or sickness caused by the LPS challenge. The decreased FI may partly be associated with the observed poor FCR and BWG. Furthermore, the presence of LPS antigen has been reported to cause excessive increase in the immune response of broilers in an attempt to combat and eliminate the presence of the foreign or exogenous threat (Xie et al., 2000; Korver, 2006). This excessive activation of the broiler immune system in response to an invading LPS antigen has been reported to have a detrimental effect on other metabolic processes aimed at normal FI, growth, and development of the bird (Hu et al. 2011). Compared to the NC, the addition of WY, YCW, YM, ZNB, and SAL to birds challenged with LPS resulted to an improvement in BWG and FCR. The reason behind this improvement when yeast and its component is supplemented to the diets of birds challenged with LPS could be associated with their ability to eliminate the cells of LPS antigens by providing competitive binding sites for these antigens. The supplements may also exhibit adjuvant-like characteristics that assist the birds to exhibit quicker and a more efficacious innate immune response in the presence of exogenous LPS antigens. The present finding is in agreement with the observation of Morales-Lopez and Brufau (2013), who reported on the immune-modulatory ability of yeast cell wall to improve the body weight and FCR of *E. coli* LPS-challenged broiler chickens.

One benefit of using in-feed antibiotics is their ability to maintain flock uniformity. The administration of LPS in the study reduced flock uniformity, similar to the observations of a previous study by Silva et al. (2015), who reported that *Salmonella* and *Mycoplasma* challenge led to a decrease in poultry flock uniformity compared to unchallenged birds. However, supplementation of the diet of LPS-challenged broilers with WY, YCW, ZNB, and SAL had similar flock uniformity to broilers in the PC group but a superior flock uniformity compared to broilers in the NC group on day 28.

The spleen and the bursa are important immune organs in birds (Xie et al., 2000; Dailey, 2002). Both immune organs help to fight certain kinds of bacteria through different immune response mechanisms (Dailey, 2002). On day 24 (post LPS administration) of the present study, the weight of the spleen and bursa had increased and decreased, respectively, in response to LPS challenge in the NC group. The increase in the relative weight of spleen of broilers in the NC group (challenged birds fed diet without any of the test additives) may be due to the occurrence of hyperplasia caused by the presence of LPS antigen that led to the activation of inflammatory cells with a resultant inflammation. This observation is in agreement with the findings of Goraca et al. (2013), who also reported an increase in spleen weight in animals challenged with LPS compared to the control group. There is little or no research that has reported the effect of prebiotic (autolyzed) yeast and its derivatives on the relative spleen weight of broilers challenged with LPS. However, a similar ameliorating effect on the spleen weight of heat stress-challenged broilers fed diets supplemented with polysaccharide from *Atractylodes macrocephala Koidz* was reported by Xu et al. (2014).

On the other hand, the decreased in bursa weight observed on day 24 of the present study could be associated with the immune response of the bursa to the LPS antigen or stress factors that may have led to marked stress, disrupted histo-morphological organization, or integrity, as well as pro-apoptotic factors that cause apoptosis and chronic atrophy of the bursa. These activities tend to reduce cellular proliferation and hence reduced bursa weight (Xie et al., 2000). The reduced bursa weight observed in broilers not receiving any of the test additives, but that were challenged with LPS (NC group) compared to all other treatment groups in the present study is consistent with the findings of Xie et al. (2000) and Ansari et al. (2017), that also reported that LPS challenge markedly reduced bursal weight in LPS-challenged broiler chickens compared to the control group. At the end of the experiment (day 35), there was no observed difference in the relative weight of the spleen, bursa, and liver between the different treatment groups. The reason behind this observation is not clear, however, it can be speculated that there is a wane in the LPS effect by natural immune response with time as the birds aged.

The increased WBC, LYM, and MONO counts observed in the blood of broilers in the NC birds may have occurred due to un-mediated natural immune response to the presence of the LPS antigens aimed at combating the invading LPS antigen. This sort of response tends to cause physiological stress on the birds, which may negatively affect some metabolic and physiological activities that may result to poor performance (Takahashi et al., 1997; Morales-Lopez and Brufau, 2013). A similar increase in the content of LYM, MONO, WBC and RBC in the blood of chickens challenged with LPS was also noticed by Xie et al. (2000). In the present study, the increase in LYM, MONO, WBC, and RBC was modulated by the presence of the additives tested, thus preventing the negative effect (compromised metabolic and physiological functions) as a result of an excessive immune response (the LPS challenge) may have caused (Morales-Lopez and Brufau, 2013). On day 24, albumin and IgG increased while IgM decreased in response to LPS challenge. This could be as a result of the response of the immune organs and defence mechanism to combat the invading LPS antigen. According to several reports, this mobilization or increased immune response, if not regulated, can have a negative effect on other metabolic processes such as growth performance (Xie et al., 2000; Brzek and Konarzewski, 2007). According to Rathnapraba et al. (2007), IgM is the first antibody observed during the first week post-infection in chickens. However, in a bid to decrease or counter the antigen level or effect, the IgM level may also be depleted or reduced as it encounters the antigens. Therefore, the decreased natural blood IgM concentration observed in the present trial could be associated with the interaction with the LPS antigen. However, the addition of WY, YCW, YG, YM, and ZNB, but not SAL, moderated the increase in albumin and IgG observed in the NC group. Furthermore, addition of all yeast products as well as antibiotics improved the IgM concentration compared to broilers in the NC group. This response could be a result of the ability of WY, YCW, YG, YM, and ZNB (through different mechanisms) to moderate or maintain the immune response to a level such that it successfully decreased the LPS effect without compromising other processes such as growth. This may be the reason for the improved BWG observed in the LPS-challenged groups receiving WY, YCW, YG, YM, and ZNB compared to the NC group. This result is in agreement with the findings of Paryad and Mahmoudi (2008), who reported that dietary inclusion of yeast (*Saccharomyces cerevisiae*) in broiler diets increased serum concentrations of protein and globulin. In contrast, Indresh et al. (2018), noticed that yeast nucleotides, which were present in the whole dead yeast tested in this trial, did not have a significant effect on total protein, albumin, globulin, and immunoglobulin of broilers. The contradictions observed could be due to differences in diets, health status and type and dosage of the yeast additives used, and environmental conditions.

Challenge with LPS led to a decrease in percentage carcass and meat yield (breast, thigh, and drumstick weight) among chicks in the NC group compared to chicks in the PC group on day 35 of age. The decrease in percentage carcass and meat yield noticed in the NC group correlated with the decreased FI and growth rate. Similar observations have been reported by Koh et al. (1996) as well as Laugero and Moberg (2000). However, the addition of WY, YCW, YM, YG, ZNB, and SAL to the diet of broilers challenged with LPS all led to a restoration of lost performance observed in the NC group with respect to percentage carcass and all the meat cuts considered in this study. This finding is in agreement with the observation of Fathi et al. (2012), who reported that feeding yeast culture resulted to similar improvement in carcass and meat yield of broiler chicks under immune stress when challenged with Newcastle disease virus.

## CONCLUSION

The current study confirms that LPS challenge can result in immunological stress which can negatively affect several parameters such as FI, BWG, FCR, spleen and bursa weight, flock uniformity, blood parameters, and carcass and meat yield of broiler chickens. Furthermore, this study also demonstrates that prebiotic whole yeast, yeast cell wall, yeast glucan, and yeast mannan, as well as the antibiotic additives used, through adequate adjuvant-like and immune hemostasis actions, assisted in ameliorating the stress effect caused by LPS challenge on most of the parameters considered. It can thus be suggested that, due to their similar growth supporting effect and performance relative to the antibiotic groups, the test yeast products could serve as a possible alternative to in-feed antibiotics for broiler chickens under mild challenge. Further studies should examine the efficacy of these yeast products on actual mild disease conditions such as those caused by *Clostridium perfringens* under a controlled environment.
